# Emerging of new *bioartificial* corticospinal motor synergies using a robotic additional thumb

**DOI:** 10.1038/s41598-021-97876-2

**Published:** 2021-09-16

**Authors:** Simone Rossi, Gionata Salvietti, Francesco Neri, Sara M. Romanella, Alessandra Cinti, Corrado Sinigaglia, Monica Ulivelli, Tommaso Lisini Baldi, Emiliano Santarnecchi, Domenico Prattichizzo

**Affiliations:** 1 Siena Brain Investigation and Neuromodulation Lab (Si-BIN Lab), Unit of Neurology and Clinical Neurophysiology, Department of Medicine, Surgery and Neuroscience, University of Siena, Policlinico Le Scotte, Viale Bracci, 53100 Siena, Italy; 2 Department of Information Engineering and Mathematics, University of Siena, Siena, Italy; 3 Department of Philosophy, University of Milan, Milan, Italy; 4 Cognition in Action Unit, PhiLab, University of Milan, Milan, Italy; 5 Division of Cognitive Neurology, Department of Neurology, Berenson-Allen Center for Noninvasive Brain Stimulation, Beth Israel Deaconess Medical Center, Harvard Medical School, Boston, MA USA

**Keywords:** Neuroscience, Motor control

## Abstract

It is likely that when using an artificially augmented hand with six fingers, the natural five plus a robotic one, corticospinal motor synergies controlling grasping actions might be different. However, no direct neurophysiological evidence for this reasonable assumption is available yet. We used transcranial magnetic stimulation of the primary motor cortex to directly address this issue during motor imagery of objects’ grasping actions performed with or without the Soft Sixth Finger (SSF). The SSF is a wearable robotic additional thumb patented for helping patients with hand paresis and inherent loss of thumb opposition abilities. To this aim, we capitalized from the solid notion that neural circuits and mechanisms underlying motor imagery overlap those of physiological voluntary actions. After a few minutes of training, healthy humans wearing the SSF rapidly reshaped the pattern of corticospinal outputs towards forearm and hand muscles governing imagined grasping actions of different objects, suggesting the possibility that the extra finger might rapidly be encoded into the user’s body schema, which is integral part of the frontal-parietal grasping network. Such neural signatures might explain how the motor system of human beings is open to very quickly welcoming emerging augmentative bioartificial corticospinal grasping strategies. Such an ability might represent the functional substrate of a final common pathway the brain might count on towards new interactions with the surrounding objects within the peripersonal space. Findings provide a neurophysiological framework for implementing augmentative robotic tools in humans and for the exploitation of the SSF in conceptually new rehabilitation settings.

## Introduction

Wearable robotics is an emerging field bridging engineering with neuroscience, with great translational impact into neurological and rehabilitative practice^1,2^. While many technologically advanced prosthetic devices are available for amputees, patients that still have their hand but cannot use it due to motor impairments (or paresis) of various etiologies, cannot benefit from these devices for obvious reasons. Hence, alternative strategies are needed: exoskeletons^3^ are hardly usable in activities of daily living because of their excessive weight, cumbersome size, limited wearability and lack of accommodating individual anatomical variations due to the motor impairment at the hand, forearm and upper limb. The Soft Sixth Finger (SSF), a wearable robotic additional thumb resting on the wrist that can be activated on user’s demand for grasping actions^4,5^, represents a novel augmentative/compensatory device that has been favorably accepted by post-stroke patients. Such patients, after having lost grasping abilities due to the paresis, experienced immediate functional gains in recovery of grasping and bimanual cooperation^5^. Some preliminary behavioral evidence suggests that an extra thumb might be incorporated into the user’s body schema^6^, but the brain mechanisms underpinnings SSF use are still basically unknown: healthy subjects scanned by functional magnetic resonance imaging (fMRI) during the use of SSF showed increased activations in brain regions relevant for body space and motor control^4^, but whether they have been using extant or alternative -newly formed- corticospinal motor synergies to control the hand actions performed with the supernumerary thumb is still unexplored. Understanding these mechanisms is a necessary, still unmet, step before the exploitation of supernumerary fingers in rehabilitation or augmentative scenarios.

Neural assemblies within the primary motor cortex (M1) are connected in a complex way to the periphery of the musculoskeletal system and finely tune corticospinal commands for arm and/or hand movements that require a defined coordinated activation of specific muscles and less activation, up to inhibition, of others. Therefore, M1 exerts its control of movements in terms of goal-directed actions rather than independently on single muscles. Actions are subtended by motor synergies, i.e. “the output patterns of conjoined muscle activity whose timing and amplitude modulation enable the correct production of goal-directed movements”^7,8^. Few evidences point out that, in humans and non-human primates, motor synergies are implemented within the corticospinal output: in a study combining electromyographic, kinematic and neuroimaging recordings, synergies governing several hand grasping gestures were successfully predicted by neural activation patterns within M1^9^, thus paralleling experimental results in rhesus macaques, in which the electrical microstimulation of M1 evokes complex and highly coordinated movements across multiple joints, resembling common gestures of the monkey's natural actions’ repertoire^10,11^. A similar organization can be observed in healthy humans thanks to transcranial magnetic stimulation (TMS), which is a primer^12^ for assessing corticospinal function non-invasively: for example, it has been shown that TMS-evoked finger movements after stimulation of M1 overlap those derived from grasping movements^13^.

TMS investigations have consistently revealed the engagement of the motor system in planned^14^ or executed actions^15^ capitalizing from corticospinal output changes accompanying these motor tasks resulting from the functional cooperation of M1 and the connected brain regions^16^. In healthy humans, TMS and neuroimaging investigations converge on the notion that neural networks underpinning imagined and executed actions largely overlap and functionally engage M1 as a final effector area^17–21^. The different degree of engagement of forearm and hand muscles within the motor plan dispatched, but not executed, during motor imagery can also be disentangled by TMS over M1^17,22,23^. Notably, TMS during motor imagery is more likely of capturing fine modulations of M1 activation patterns than during overt movements, when the whole corticospinal system is operating well above its threshold levels for activation, thus overshadowing local changes for synergies generation.

On these premises, we reasoned that the pattern of TMS-evoked responses in different muscle groups of the forearm/hand during imagined grasping actions of objects could represent the most appropriate approach to verify the specific hypothesis of the study, that was to reveal different motor synergies eventually recruited when using the additional SSF versus naturally performed actions. Understanding mechanisms of these *bioartificial synergies* is a crucial step before extra fingers (or arms) could be fully exploited into rehabilitation settings for compensation, as well as in forthcoming scenarios of augmentation of physiological human capabilities, that are not so far away to come^24^.

## Methods

### The soft sixth finger (SSF)

**Figure 1:**
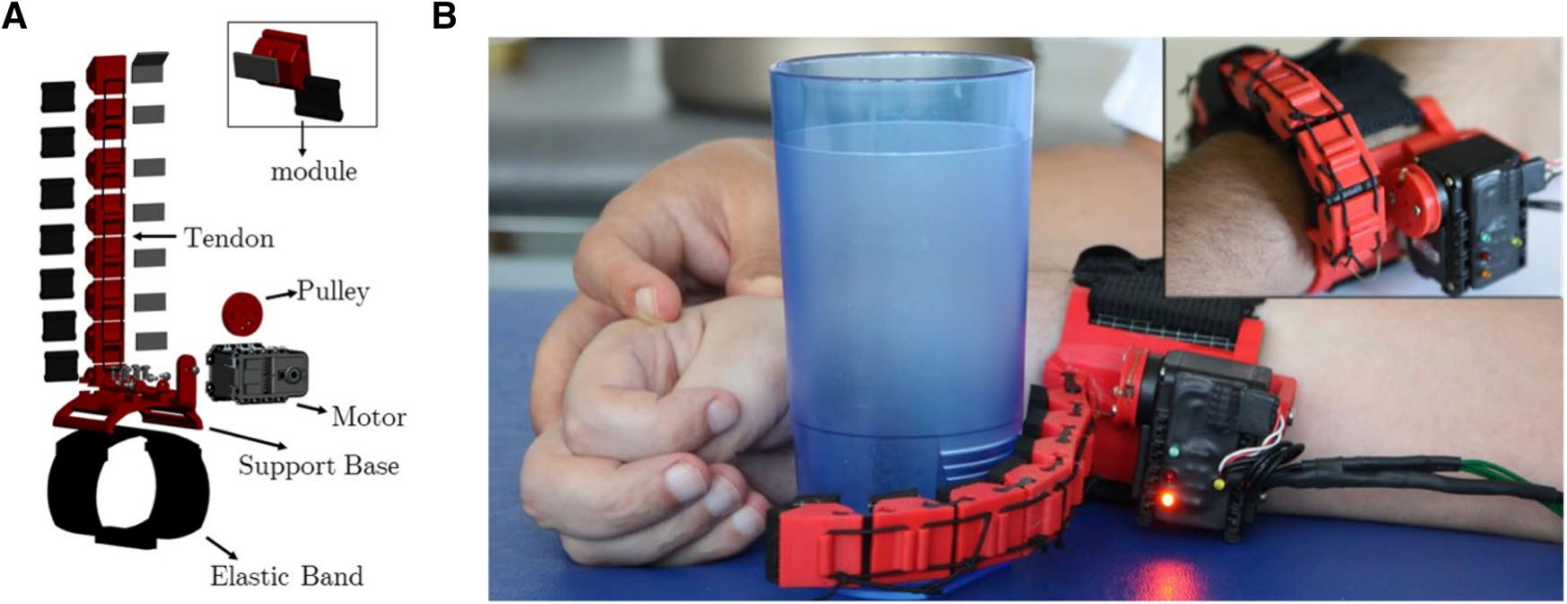
The Soft Sixth Finger. (**A**) The SSF has a modular structure. Each module is consist of a rigid ABS part and a soft Polyurethane part that acts like a soft joint. The finger is actuated by a tendon-driven system with a single motor. (**B**) Upper right corner: the finger can be worn at the forearm and can be wrapped around the wrist when not used. When in use, the SSF acts as a gripper with the rest of the hand (in this case a paretic hand is showed). [Original photographs taken by Gionata Salvetti].

The SSF (Fig. 1) is a 140 g weighing robotic wearable robotic supernumerary extra finger that acts as an additional thumb^4,5,25^. As the SSF does not rely on subject’s skeletal structure, anatomical variability and motion restriction are a minor issue. It represents the minimal complexity solution for grasping that also guarantees extreme wearability. After being fasten on the wrist by means of an elastic yet stable band, the system composed of the SSF and the hand/forearm acts like a gripper to hold and manipulate an object. The SSF can stably grasp objects of different shape and size thanks to its intrinsically compliant structure. The device can be used on demand through different kinds of system interfaces^26^ while resting wrapped around the wrist, as a bracelet, when not in use.

### Participants, task description and experimental paradigm

Thirty (twenty-seven fully right-handed) healthy subjects (male: 15; female 15; mean age 29.5 ± 7.5 SD years) participated in the study after having signed an informed consent and having received the approval of the study by the Local Ethic Committee [Ethic Committee Regione Toscana, Area Vasta Sud-Est (CEAVSE)]. All experiments were carried out in accordance with the ICH Guideline for Good Clinical Practice.

The sample size was estimated expecting as significant an amplitude variation of 25%, with an α error probability of 0.05, nonsphericity correction ɛ = 1,within an ANOVA for repeated measures, according to the experimental design (one group of healthy subjects, five conditions): output parameters sorted a sample of 26 subjects to achieve a statistical power of 0.90 and to discriminate significant changes between different conditions (Noncentrality parameter λ: 16.25; Critical F: 2.46; degrees of freedom: 4).

Handedness was assessed by Oldfield’s questionnaire^27^. All subjects had no contraindications to TMS^28^, were free from neurological or psychiatric disorders, did not assume drugs for therapeutic purposes and had a quite normal sleeping night the day before the experiment. None declared the assumption of recreative substances in the last ten days.

**Figure 2:**
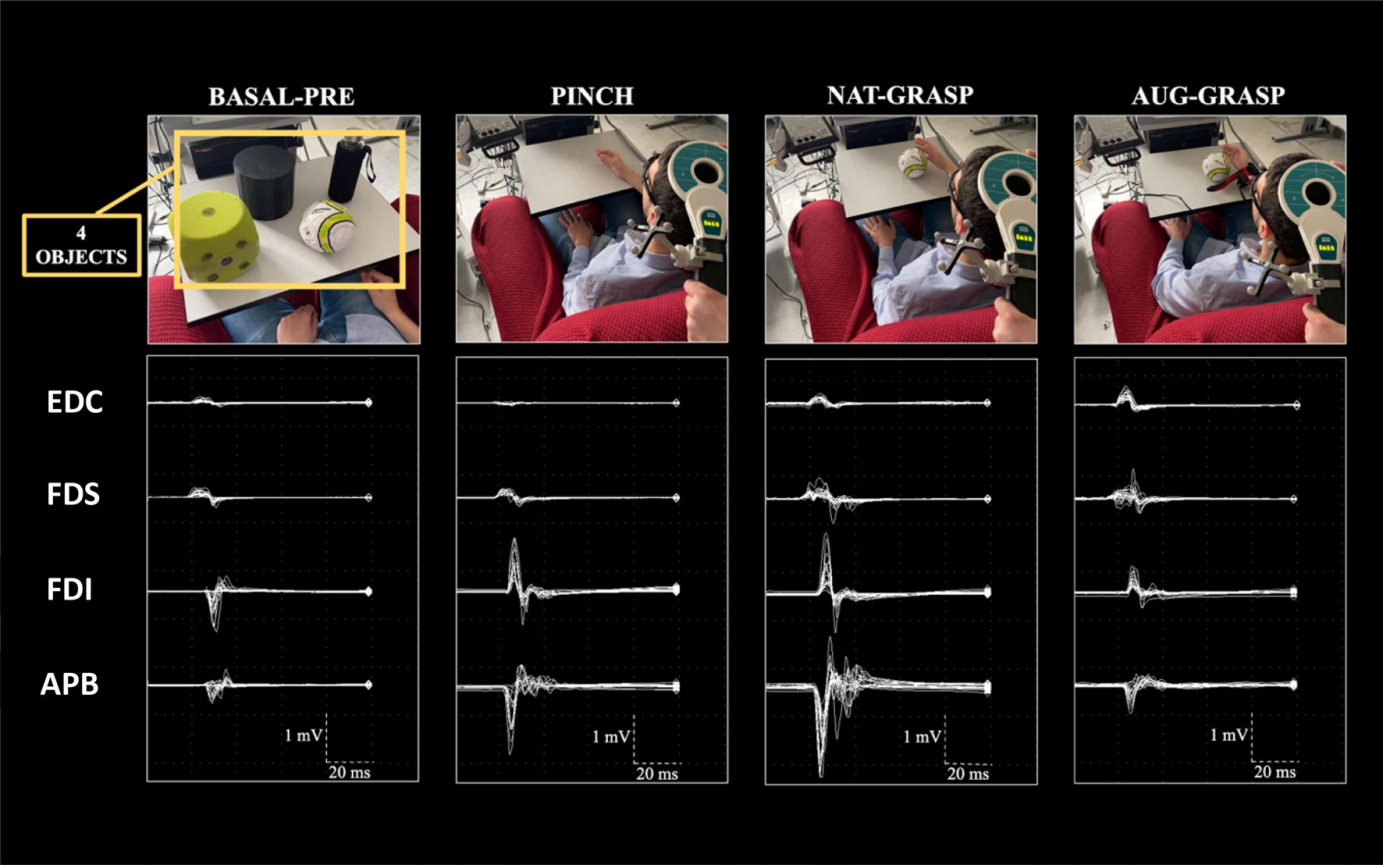
Experimental conditions. MEPs were simultaneously recorded from Extensor Digitorum Communis (EDC), Flexor Digitorum Superficialis (FDS), Abductor Pollicis Brevis (APB) and First Dorsal Interosseous (FDI) muscles during resting condition (BASAL-PRE), imagery of a pinch-grip (PINCH) between the thumb and index finger, imagery of a natural grasping (NAT-GRASP) or augmented grasping (AUG-GRASP). MEPs recorded during Basal-post condition are not shown. [Original photographs of the experimental setup taken by Simone Rossi].

The main task consisted in performing the mental imagination of grasping actions with or without the SSF. Objects to be grasped were a bottle, a cylinder, a ball, and a foam rubber dice (mean weight: 262.5 ± 116.1 g), without particular affordances able to influence the grasping pre-shaping at cortical level. Participants sat in front of a table where one of the four objects was laying, with their arm resting near it (Fig. 2). All subjects were given the opportunity to train with the grasping tasks and with the SSF use before starting the experiment, until they were able to complete a true grasping action with the SSF and stated to be confident in its use. Then, subjects were trained to perform the imagery task of grasping without producing any electromyographic activity in the four recorded muscles (flexors and extensors at the forearm and two intrinsic muscles of the hand, see Fig. 2). This two-step training required no more than three minutes.

After having located the left motor cortex on the scalp and having found the individual threshold of stimulation for TMS (see the following paragraph), the following experimental conditions were considered:

1. *Basal-pre* [20 motor evoked potentials (MEPs) simultaneously recorded from the four muscles], in which subjects were instructed to fully relax, fixing a point in front of them.
2. 20 MEPs simultaneously recorded from the four muscles during motor imagery of grasping actions of the four objects (5 MEPs for each object) [Natural grasping (*Nat-grasp*)].
3. 20 MEPs simultaneously recorded from the four muscles during motor imagery of grasping actions of the four objects (5 MEPs for each object) with the SSF [Augmented grasping (*Aug-grasp*)].
  The participants wear the SSF in this grasping condition only.
4. 20 MEPs from the four muscles during motor imagery of a pinch-to-grip-action involving the index finger and the thumb (*Pinch*).
5. 20 MEPs simultaneously recorded from the four muscles in a complete rest condition, wearing the SSF (*Basal-post*).

Conditions 2, 3, and 4 were balanced and randomized between subjects. In conditions 2 and 3, the four objects were randomly presented to the subject, in successive blocks of five trials each. Multiple objects (without definite affordances) were used to simulate an ecological use of the SSF in daily living, where a person with reduced hand movement abilities must interface to various items in terms of shape, compliance and weight. Moreover, the secondary aim was to avoid an habituation effect and a flattening an electrophysiological response caused to a multiple presentation of the same object.

In all motor imagery conditions, the participants imagined the grasping movement they should have done, but without actually doing it, starting at an acoustic “go” signal given by one of the experimenters. The subject was required to imagine the movement in its entirety, from the rest position to the final grasp of the object, but without lifting it. The TMS pulse randomly followed 1–3 s the “go” signal, therefore occurring during the reaching phase, and a random time between 5 and 10 s elapsed between two consecutive "go" signals, aiming to minimize expectancy effects^17,21,23^. This time served also to monitor the electromyographic (EMG) silence in the target muscles in the time window preceding the brain stimulation. The EMG silence preceding the TMS pulse was also monitored using an acoustic feedback provided by the EMG recorder. The whole experimental paradigm, that lasted between 45 min and one hour, was designed for being as ecological as possible (i.e., as close as possible to everyday life). Figure 2 shows representative MEPs recorded in one of the subjects.

### Procedures of brain stimulation and neurophysiological recording

A circular non-focal 90-mm coil connected with a ATES magnetic stimulator (EBNeuro, Italy) was positioned over the vertex with its handle pointing backwards, so that the electric field flowed in a posterior-anterior counterclockwise direction. The cortical representations of the right Extensor Digitorum Communis (EDC), Flexor Digitorum Superficialis (FDS), Abductor Pollicis Brevis (APB) and First Dorsal Interosseous (FDI) were targeted within the left M1. The use of a non-focal coil guaranteed fairly stable simultaneous MEPs from all the considered forearm/hand muscles, even if the coil did not cover evenly the cortical hot-spots of the four muscles. To the same aim, the intensity of each TMS pulse was adjusted to obtain fairly stable MEPs simultaneously from the four muscles (mean 113%, SD 2.9, of the resting motor threshold, corresponding to the 55–75% of the maximal stimulator output among all subjects), defined as the minimal intensity to produce a MEP of 50–100 µV in the APB muscle with a probability of 50%^29^.

Once identified the best fitting scalp position for the coil for targeting M1, this was maintained throughout the experiment by means of a neuronavigation system (BrainNET, EBNeuro Ltd, Florence, Italy) using infrared cameras (Polaris Vicra, NDI, Waterloo, Canada). An MRI template for each participant was uploaded in the neuronavigation software and a co-registration procedure was performed using scalp landmarks (nasion, vertex and the two preauricular points) and additional landmarks positioned on a plastic glasses frame worn by the subjects. The coil was calibrated using an in-house algorithm based on five landmarks specific for the device. During TMS, the software provided online visual feedback allowing the investigators to keep constant (with a tolerance of 2 mm for each spatial x, y, z axis) the desired coil orientation/rotation/distance during the whole session.

Corticospinal responses (or MEPs) were recorded by means of surface electrodes placed with a belly/negative-tendon/positive montage; analysis time base was 50–100 ms, bandpass acquisition filers were 20–20.000 Hz, the gain of amplifiers was adjusted to entirely show the negative–positive evoked peaks of MEPs, without saturation.

### Neurophysiological data analysis

#### Pre-processing

For each of the four muscles, the peak-to-peak amplitude of the MEPs obtained in each experimental condition were considered. Trials with detectable voluntary EMG activity in the analysis time were discarded from further analysis. Whenever unwanted EMG or other artifactual activity contaminated one of the 4 MEPs simultaneously acquired for each TMS pulse, or if there was a latency jitter of more than 3 ms in one of the four MEPs, all the remaining responses were discarded as well, even if they were artifact-free. Outliers in amplitude (i.e., more than 2 standard deviations from the average value of the considered condition) were also discarded. The final rejection rate ranged 10–20% (summing rejections during the acquisition phase and post-experimental visual inspection) between subjects. After these procedures, a sample of at least 15–18 MEPs/condition remained available for successive processing.

### Statistical analysis

Data were analyzed using SPSS Version 20 statistic software package (IBM Corp. Released 2011. IBM SPSS Statistics for Windows, Version 20.0. Armonk, NY: IBM Corp). Since the assumption of normality as assessed by the Shapiro–Wilk test was violated (*p* < 0.05), nonparametric Friedman test statistics were adopted for the repeated measures experimental design addressing amplitude changes across conditions.

As a first step, the modulation of MEPs amplitude of each muscle (APB, FDI, FDS, EDC) was separately investigated across the five conditions (*Basal-Pre, Nat-grasp, Aug-grasp, Pinch, Basal-post*). Three outliers (mean amplitude of participant’s MEPs higher than 2 SD compared to the group average) for APB were excluded. The statistical analysis for this muscle was thus conducted on twenty seven healthy subjects (male 14; female 13).

A second test was carried out to investigate differences of the MEP amplitudes by coupling responses from the intrinsic hand (APB and FDI) and extrinsic forearm hand muscles (FDS and EDC) across conditions (5 levels: *Basal-pre, Nat-grasp, Aug-grasp, Pinch, Basal-post*). Here, by means of a preliminary analysis of the average values within each condition, one outlier was identified (mean amplitude of participant’s MEPs higher than 2 SD compared to the group average), and thus excluded from the analysis. The statistical analysis was thus conducted on twenty-nine subjects (male: 14; female 15).

Finally, a third test was performed to evaluate modifications of MEPs amplitude between agonist (FDI and FDS) and no-agonist (APB and EDC) muscles in the imagined actions. Amplitude variation of these two groups of muscles were compared (factor Condition: 5 levels: *Basal-pre, Nat-grasp, Aug-grasp, Pinch, Basal-post*). As the preliminary analysis identified one outlier (mean amplitude of participant’s MEPs higher than 2 SD compared to the group average), it was excluded from the analysis, that was conducted on twenty-nine subjects (male: 15; female 14).

Whenever the Friedman’s test resulted significant, Dunn’s pairwise post-hoc tests were conducted, followed by Bonferroni correction for multiple comparisons. For all tests, the level of significance was set at *p* < 0.05. While statistics were carried out on raw MEPs amplitude values, also percentage variations were used for graphical representations (see Supplemental Material) by considering the *Basal-pre* condition as 100%, irrespective of the absolute average value of MEP amplitudes.

These analyses allowed to verify modifications of the specific corticospinal output independently for each muscle, as well as for distinct corticospinal synergy patterns across the different conditions of movement imagination.

All statistical comparisons are available in the Table S2 of the supplemental material, as well as graphical representations of percentage variations (Figure S1, S2 and S3).

### Significance statement

The study shows the emergence of new bioartificial cortico-muscular synergies, as revealed by direct stimulation of the primary motor cortex by transcranial magnetic stimulation, induced by a wearable robotic sixth finger designed for augmentation of grasping in healthy humans and for recovery of grasping in paretic patients.

## Results

Table S1 reports MEPs’ mean amplitude values and standard deviations in each individual muscle in the basal condition. Table S2 reports all statistics. Figures S1-S3 additionally show percentual amplitude data variations in all conditions.

### Single muscles level

**Figure 3:**
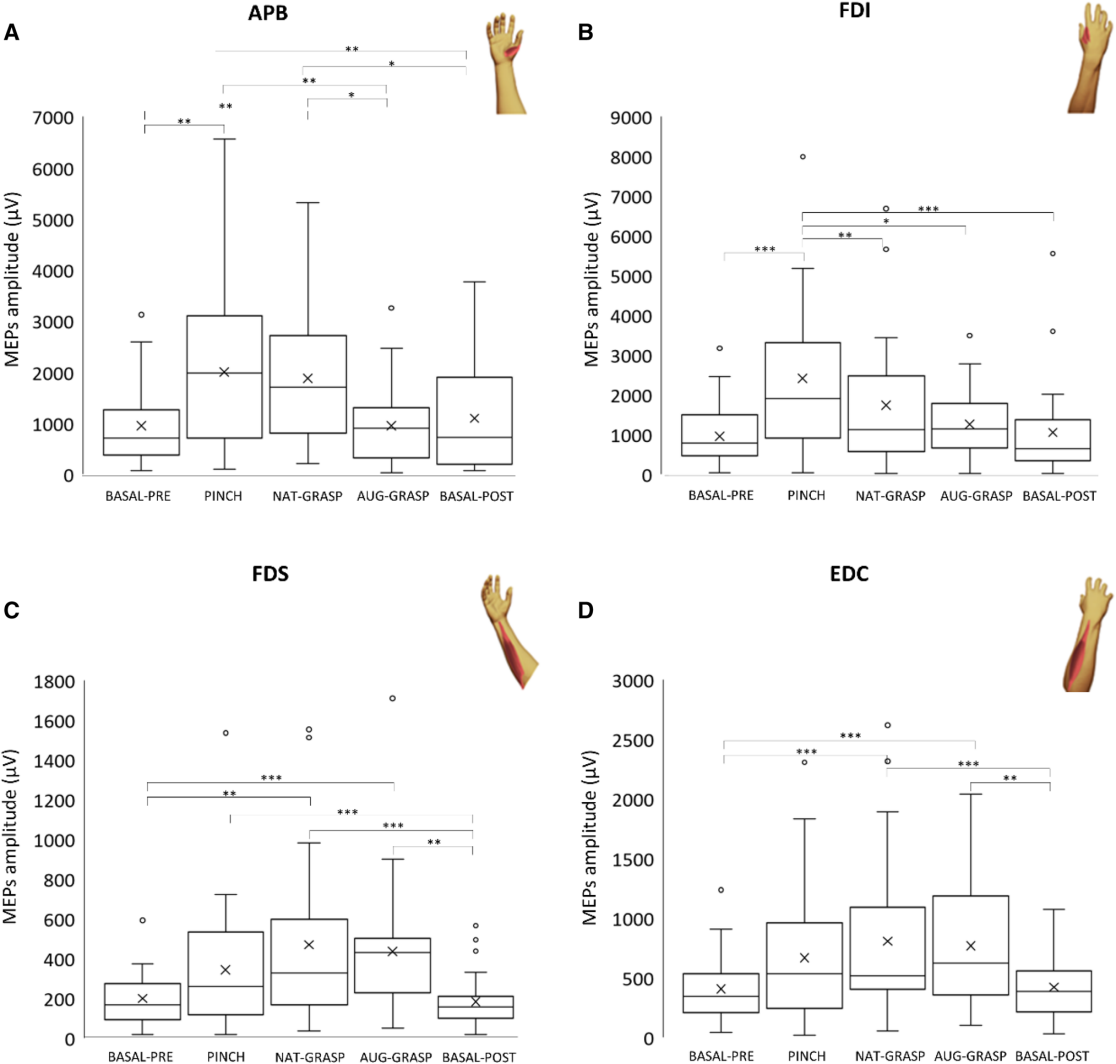
MEP amplitude changes at single-muscle level. (**A**) APB variation versus resting condition (BASAL-PRE); (**B**) FDI variation versus resting condition; (**C**) FDS variation versus resting condition; (**D**) EDC variation versus resting condition. Here and in the following figures, the following parameters of raw amplitude data distribution are indicated: mean and median values (the cross and the line within the box), the 25th and 75th quartiles (the borders of the box), the 5th and 95th percentiles (the extremes of the whiskers). Small circles indicate outliers.

Figure 3 shows the variation of MEPs amplitude versus *Baseline-pre* in the different conditions. Table S2 reports all statistics. Concerning APB MEPs, a significant difference across conditions (χ^2^_(4)_ = 28.28; *p* < 0.001) emerged. Post hoc tests revealed a significant increase of the MEPs amplitude in the conditions *Pinch* (*p* < 0.001) and *Nat-grasp* (*p* = 0.002) compared to *Basal-pre*. The condition *Nat-grasp* differed significantly also from *Post* (*p* = 0.019), while *Aug-Grasp* differed significantly from *Nat-grasp* (*p* = 0.048) and from *Pinch* (*p* = 0.019). Finally, the MEPs amplitude in *Basal-post* condition significantly differed from *Pinch* (*p* = 0.007) and *Nat-grasp* (*p* = 0.019) conditions, but not from *Aug-grasp.*

A significant difference across conditions (χ^2^_(4)_ = 34.528; *p* < 0.001) occurred also for FDI MEPs: post-hoc tests showed a significant increase of the MEP in the condition *Pinch* versus *Basal-pre, Post, Aug-grasp and Nat-grasp* (*p* < 0.001, *p* < 0.002, *p* = 0.043 and *p* = 0.003 respectively).

Differently, for FDS MEPs (χ^2^_(4)_ = 44.614; *p* < 0.001), the MEPs amplitude of *Basal-pre* condition significantly increased during both *Nat-grasp* and *Aug-grasp* (respectively: *p* < 0.006 and *p* < 0.001); MEP amplitude significantly increased in *Nat-grasp* and *Aug-grasp* compared to the *Basal-post* (both *p* < 0.001). Additionally, the MEPs amplitude of *Pinch* condition was higher (*p* = 0.005) compared to that of *Basal-post*.

A similar corticospinal modulation was observed for EDC MEPs (χ^2^_(4)_ = 32.514; *p* < 0.001), whose variation versus the MEPs amplitude of the *Basal-pre* condition significantly increased during *Nat-grasp* and *Aug-grasp* (both *p* < 0.001). Again, it was significantly augmented in *Nat-grasp* and *Aug-grasp* compared to the *Basal-post* (both *p* < 0.001).

Taken together, this first analysis at single-muscle level (Fig. 3) showed that the FDI muscle was involved only in the *Pinch* condition, the APB muscle was involved in the *Pinch* and in the *Nat-grasp*, but not in the *Aug-grasp* (i.e., when its function was replaced by the SSF), while the two forearm muscles were involved both in the *Nat-grasp* and *Aug-grasp*, but not in the precision pinch grip. MEPs amplitude between *Basal-Pre* and *Basal-post* conditions did not significantly differ for any muscle.

### Intrinsic (“Hand”) versus extrinsic (“Forearm”) hand muscles

**Figure 4:**
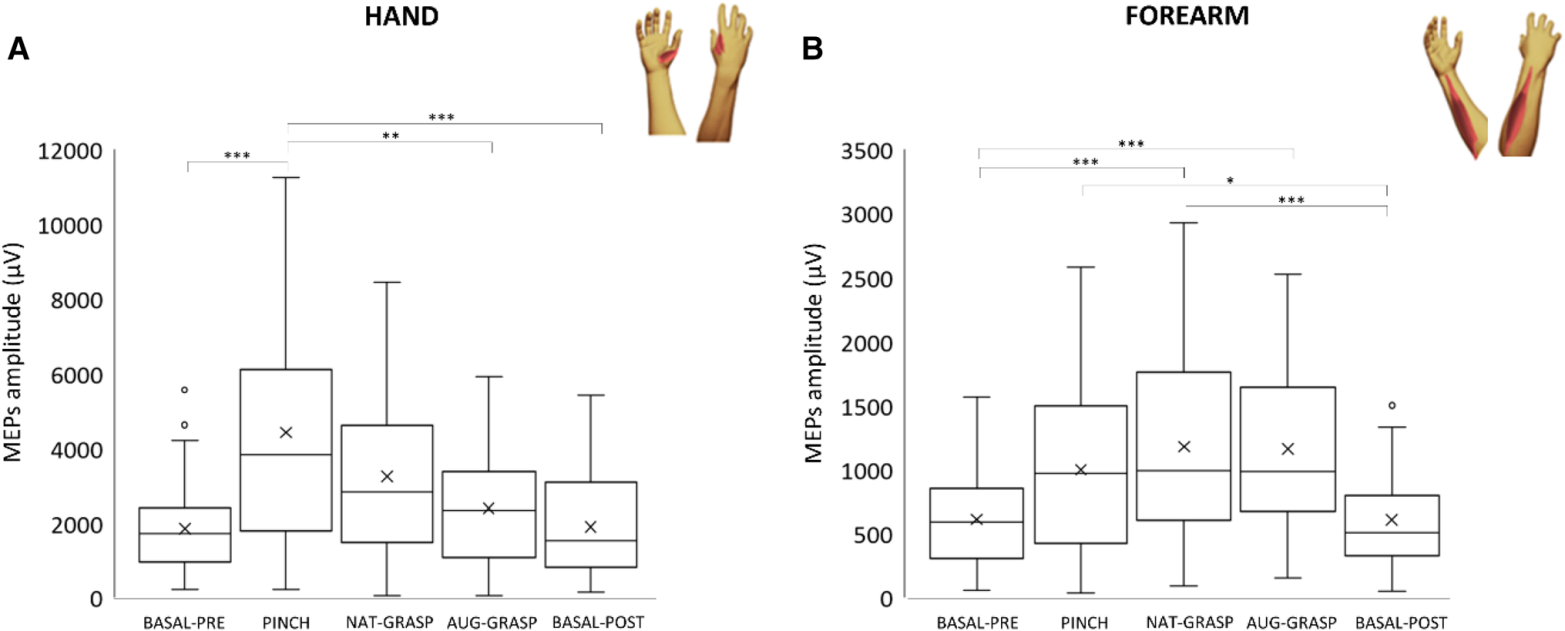
Intrinsic (“Hand”) versus extrinsic (“Forearm”) muscles effects. MEPs amplitude modulation versus Basal-Pre condition of distal HAND (FDI + APB) versus proximal FOREARM (EDC + FDS) muscles. Same organization as the previous figure.

Figure 4 shows “Hand” and “Forearm” MEPs amplitude variation versus *Baseline-pre* in all experimental conditions; Table S2 reports all statistics.

Analyzing the “Hand” MEPs amplitude, a main effect of Condition was found (χ^2^_(4)_ = 33.112; *p* < 0.001), the corticospinal output for the intrinsic hand muscles being significantly increased (*p* < 0.001) versus *Basal-Pre*, only during the imagery of the pinch grip. Considering comparisons between the other conditions, MEPs amplitude was higher at *Pinch* compared to *Basal-post* and to *Aug-Grasp* (*p* < 0.001 and *p* = 0.002). No other significant variations were observed.

Considering the extrinsic hand “Forearm” muscles, a significant main effect of Condition was detected (χ^2^_(4)_ = 39.965; *p* < 0.001). Post hoc tests revealed that the corticospinal output for extrinsic forearm muscles significantly increased during the *Nat-grasp* (*p* < 0.001) and *Aug-grasp* (*p* < 0.001) conditions compared to *Basal-pre* and *Basal-post*, but not during *Pinch*. MEPs amplitude was higher during the *Nat-grasp*, *Aug-grasp* and *Pinch* compared to *Basal-post* condition (*p* < 0.001, *p* < 0.001 and *p* = 0.024 respectively).

An opposite trend (that did not reach significance) was observed for “Forearm” and “Hand” muscles when engaged in *Nat-grasp* and *Aug-grasp*: *Aug-grasp* increased corticospinal output towards “Forearm” muscles and decreased it versus *Nat-grasp* in “Hand” muscles.

### Agonist versus no-agonist muscles

**Figure 5:**
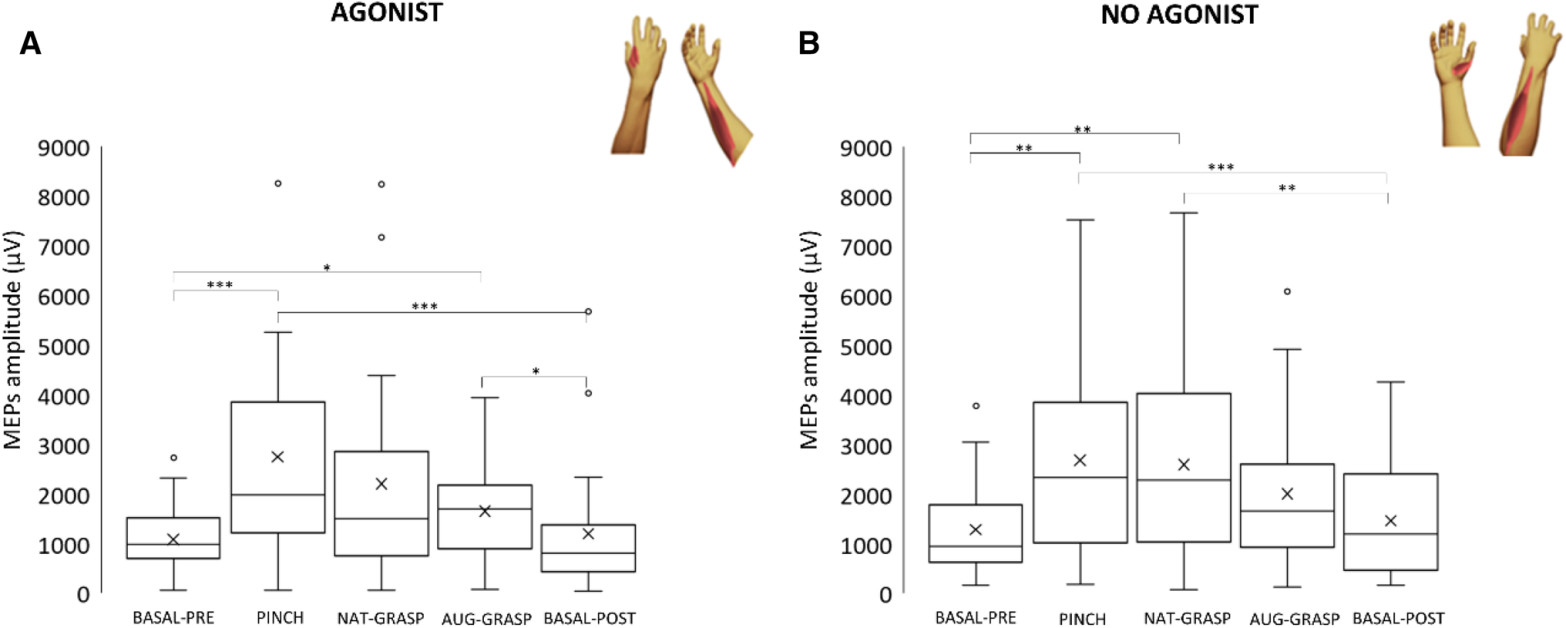
Agonist (FDS + FDI) versus non-agonist (EDC + APB) muscles MEPs amplitude. (**A**) Agonist muscles MEPs amplitude variation versus resting condition (BASAL-PRE); (**B**) Non-agonist muscles MEPs amplitude variation versus resting condition (BASAL-PRE). Same organization as previous figures.

Figure 5 shows amplitude variation of MEPs amplitude versus *Baseline-pre* in all experimental conditions; Table S2 reports all statistics.

Considering the corticospinal output in agonist muscles (FDI + FDS), a significant main effect of Condition was found (χ^2^_(4)_ = 37.492; *p* < 0.001). The MEP amplitude of these muscles significantly increased in conditions *Pinch* and *Aug-grasp* versus the MEPs value of *Basal-pre* condition (*p* < 0.001 and *p* = 0.032 respectively). Analyzing the activity of non-agonist muscles (APB + EDC), a main effect of Condition was again detected (χ^2^_(4)_ = 30.059; *p* < 0.001): post-hoc tests revealed a significant increase versus *Basal-pre* of MEPs recorded during *Pinch* (*p* = 0.001) and *Nat-grasp* (*p* = 0.005), but not versus *Aug-grasp* condition. MEPs amplitude recorded during *Pinch* and *Nat-grasp* was higher compared to those of *Basal-post* (*p* < 0.001 and *p* = 0.005 respectively).

## Discussion

As shown in two families of individuals born with six-fingered hands, a genetic condition known as polydactyly^30^, the human brain is able to control a body part with over-physiological degrees of freedom, as a supernumerary fingered hand. In these individuals, the innate supernumerary finger augments some manipulation abilities versus a conventional five-fingered hand, but it is controlled neuromechanically by independent muscles and nerves, and its somatotopy at cortical level is distinct from that of the other fingers^31^. However, brain motor synergies controlling the supernumerary finger, not addressed indeed, remain obscure. Even in conventional five-fingered living humans, “peripheral motor synergies” have been extensively investigated biomechanically, kinematically and in terms of electromyographic activity^32–34^, but very seldom addressed from a central nervous system control’s perspective^23,35–38^.

The current study is based on the unique opportunity to model the neural adaptation of healthy humans to an augmentative supernumerary thumb. Results show that using this acquired six-fingered hand, emerging bioartificial corticospinal synergies, different from natural ones, may be disclosed. Unlike individuals with polydactyly, that are “pre-wired” in this sense, these emerging synergies should obviously be implemented by the same muscle groups used for controlling natural hand actions, although recruited with different patterns of excitation/inhibition: these patterns can be reflected in the modulation of muscle responses following an invariant TMS pulse as intensity and location (i.e., a navigated supra-threshold stimulus of the contralateral M1).

This is not surprising, according to previous experimental evidences and to some established physiological notions: (1) in monkeys, a motor cortical region containing neurons coding functional synergies for proximal (extrinsic) and distal (intrinsic) muscles has been identified^39,40^; (2) within M1, neurons controlling individual hand muscles are widely represented and overlap the distribution of neurons controlling other hand muscles^41^; (3) under physiological conditions (i.e., without supernumerary fingers) the redundancy of the musculoskeletal system allows the motor system to employ a restricted set of modular commands (i.e., synergies) to accomplish both automatic and goal-directed actions^42^; (4) this strategy is aimed at reducing the dimensionality of motor commands with consequent flexibility and computational brain’s advantages. Such strategy might rely on common yet divergent cortical outputs^43^ impinged by descending volleys induced by TMS towards spinal motoneurons: these can control either extrinsic hand muscles via polysynaptic connections (with diverging commands being mediated by propriospinal interneurons^44,45^) or intrinsic ones, via more direct monosynaptic connections^46^, during imagined grasping with or without the SSF. So, despite physiological synergies are invariant and hard-wired as patterns of activation across muscle groups^11^, current results suggest that they can immediately be reshaped by adding a new and unexpected bioartificial effector within the motor plan for grasping. Such a flexibility of motor synergies, even beyond the physiological repertoire (as wearing an extra finger), represents a still unexplored capability of the human brain.

More in detail, during rehearsal of the pinch grip between index finger and thumb, the more involved muscles were the two intrinsic hand muscles (Figs. 3a, b, 4a), with a synergistic contribution of the FDS muscle at the forearm, that is agonist for the index finger displacement in flexion (Fig. 5a). Imagined actions of whole hand grasping did not significantly modulate the corticospinal drive towards hand muscles (Fig. 4a), but a clear-cut facilitation of both flexors and extensor muscles at the forearm level occurred (Figs. 3b, c, 4b), either when imagining the grasping with (*Aug-grasp*) or without the SSF (*Nat-grasp*). Crucially, during the augmented grasping, the corticospinal drive towards the APB, prime mover for the thumb, did not change versus the resting condition: this also resulted in a significant difference between *Nat-Grasp* and *Aug-grasp* (Table S2 and Fig. 1), according to the exclusion of the natural thumb from the planning of the grasping action performed with the aid of the extra finger. Finally, the two resting conditions in which the extra finger was or was not present, did not differ each other: this suggests that robotic finger was able to modify the corticospinal output only when engaged in the planning of an action to be performed.

Planning a grasping action requires the functional cooperation of a wide premotor-parietal network that identifies the target, determines the action goal and the trajectories of hand/object interactions, while coding the peripersonal space in which the forthcoming action will occur^46,47^ (for recent reviews on the topic see^48–50^). All these information finally converge into the M1 as an effector area for muscles^51^. This network is similarly active either during voluntary actions or imagery of the action itself^19^. The necessary bi-directional communication within the prefrontal-parietal network for action planning relies on associative intracortical horizontal fibers located in the most superficial layers of the cortex, known to be the ones more prone to be activated by a TMS pulse^52^ that -if slightly suprathreshold- determines the final trans-synaptic firing of the corticospinal cells and the following muscular response^53^. As a consequence, any functional change occurring in this network (either at premotor or parietal hubs or within M1 itself) will finally modulate the activity of the corticospinal system, even when activated by the TMS pulse instead by the physiological motor command. Current results show a robust and immediate modulation of corticospinal output for hand and forearm muscles in all the experimental imagery conditions (i.e., pinch grip, grasping with or without the SSF), despite these muscles are supplied by physiologically different descending systems in terms of number of synapsis they are controlled by^46^*.* Both muscles groups, however, seem to be equally susceptible to the sum of modulating converging inputs coming from the different nodes of the premotor-parietal network, suggesting that the neural computation of corticospinal synergies represents a robust top-down hierarchical mechanism governing both direct monosynaptic and indirect polysynaptic cortico-muscular connections.

Within this framework, however, it should be noted that it is exactly the phylogenetic development of the corticospinal system that has allowed the finest shaping of hand movements, as well as the acquisition of new complex hand motor skills only in those species with fully developed monosynaptic connections for hand muscles control (i.e., from non-human primates onwards)^54^. This is clearly evident by the selective modulation of FDI and APB intrinsic hand muscles during the imagination of the pinch-grip, in which they act as the main effectors of the precision action. Such a fine modulation of corticospinal output also inherently shows that subjects were indeed able to correctly perform the imagery tasks.

A large part of the premotor-parietal network for actions control in humans is devoted to the representation of the own’s body schema, a hierarchical high-level construct indicating a non-conscious process, continuously updated during movements, through which the individual registers his posture (or body part position) in relation to the peripersonal space^55^. Body schema is intrinsically highly flexible and adaptable: for example, the level of dexterity in tools use is reached once the tool has been incorporated, or embodied, into the body schema^56^ and that repeated tool use (as a mechanical grabber extending the space around the arm) may carry after effects on subsequent free-hand grasping and pointing movements, likely altering the individual’s body schema^57^. Emerging behavioural data indicate that a supernumerary finger, which is conceptually similar to a common tool, can actually be embodied into the user’s body schema^6^*,* even when presented as an avatar in virtual reality scenarios^58^. This implies that the brain may readily adapt to various configurations of the hand and to new action strategies^59^ at least when they occur within the peripersonal space. The reshaping of corticospinal motor synergies (or plasticity of motor synergies), which would hardly happen without the SSF being incorporated into the own body schema, is the neurophysiological mechanism that could allow such a flexibility.

An intriguing issue to explore in future studies is to directly test the functional connectivity between parietal or premotor hubs of the grasping network with the M1: this could be done by paired, appropriately spaced in time, TMS pulses^60,61^ delivered in concomitance with different timing of the (imagined) grasping action. Such paired TMS approach (a conditioning stimulus on premotor or parietal targets preceding the test pulse on M1), has been proven useful in verifying the role of the ventral premotor cortex in precision^60^ or object-driven^62^ grasping, of the dorsal premotor cortex in planning a grasp^63^, or in disentangling the role of different sub-regions of the parietal cortex in action planning/execution^61,64^ or movement observation^65^. In the frame of such paired-pulse paradigm, with the MEPs simultaneously recorded by several intrinsic and extrinsic upper limb muscles, it could be directly verified whether the observed changes of bioartificial motor synergies depend mainly by cortical (either premotor or parietal) activity upstream to M1, or take place directly at M1 level.

In conclusion, current results show how the human motor system is capable of quickly welcoming augmentative bioartificial corticospinal grasping strategies, in the frame of a still unexplored system-level form of brain plasticity. This ability might represent the functional substrate of a final common pathway the brain might count on towards new interactions with the peripersonal space, once the supernumerary finger has been embodied into the user’s body schema. Understanding these immediate plastic brain changes is a necessary step towards the exploitation of supernumerary fingers in conceptually new home-based rehabilitation settings for grasping compensation or reacquisition of bimanual task cooperation in patients with hand paresis (i.e., post stroke and of other origin)^5^. More generally, current results disclose the neuroscientific foundations needed for correctly approaching the emerging field of “human augmentation”, a scenario that refers to a new class of wearable technologies aimed to resemble -or even surpass- human limb functions, thereby facilitating the integration of supernumerary fingers -or even arms- with user’s natural abilities^2,24^.

## Supplementary Information

Supplementary Information.

## Data Availability

All data used to support the findings of this study are included within the article. Additional data is available upon request to the Corresponding Author.
