# Supplementary material for: Emerging of new bioartificial corticospinal motor synergies using a robotic additional thumb
Source: Sci Rep. 2021 Sep 16;11:18487. doi: 10.1038/s41598-021-97876-2 (PMC8445932; doi:10.1038/s41598-021-97876-2)

**EMERGING OF NEW *BIOARTIFICIAL* CORTICOSPINAL MOTOR SYNERGIES USING A ROBOTIC ADDITIONAL THUMB**

Simone Rossi^1,CA^, Gionata Salvietti^2^, Francesco Neri^1^, Sara M. Romanella^1^, Alessandra Cinti^1^, Corrado Sinigaglia^3,4^, Monica Ulivelli^1^, Tommaso Lisini Baldi^2^, Emiliano Santarnecchi^,5^, Domenico Prattichizzo^2^

^1^ *Department of Medicine, Surgery and Neuroscience, Unit of Neurology and Clinical Neurophysiology, Siena Brain Investigation and Neuromodulation Lab (Si-BIN Lab), University of Siena, Italy*

^2^ *Department of Information Engineering and Mathematics, University of Siena, Italy*

*^3^ Department of Philosophy, University of Milan, Milan, Italy^.^*

*^4^ Cognition in Action Unit, PhiLab, University of Milan, Milan, Italy^.^*

*^5^ Berenson-Allen Center for Noninvasive Brain Stimulation, Department of Neurology, Division of Cognitive Neurology, Beth Israel Deaconess Medical Center, Harvard Medical School, Boston, MA, USA*

CA: Simone Rossi, MD, PhD

Siena Brain Investigation & Neuromodulation Lab (Si-BIN Lab)

Department of Medicine, Surgery and Neuroscience, Unit of Neurology and Clinical Neurophysiology

University of Siena, Italy

Policlinico Le Scotte, Viale Bracci,

I-53100, Siena, Italy

E-mail: [Simone.rossi@unisi.it](mailto:Rossisimo@unisi.it)

Tel: ++39 0577 233 321

Fax: ++39 0577 270 260

www.sibinlab.com

**Conflict of interest**: All the Authors declare that there is no conflict of interest regarding the publication of this paper.

**Data availability statement**: All data used to support the findings of this study are included within the article. Additional data is available upon request to the Corresponding Author.

**Funding**: The research was partly granted by the EU Project SOFTPRO (Synergy-based Open-source Foundations and Technologies for Prosthetics and RehabilitatiOn), European Union's Horizon 2020 research and innovation program, Grant Agreement No. 688857

**Acknowledgments**: Authors thank Dr. Carmelo Smeralda, Mattia Pomes and Zubar Iqbal for experimental help, and Prof. Patrizio Pasqualetti for fruitful discussions on statistics.

1) Results

**Supplementary Table S1.** Mean and standard deviations (in parenthesis) of MEPs amplitude in the four muscles during Basal condition. Numbers are µV.

|  | **Subjects** | **APB** | **FDI** | **FDS** | **EDC** |
| --- | --- | --- | --- | --- | --- |
|  | 1 | 429 | 598 | 87 | 242 |
|  |  | *(292)* | *(312)* | *(18)* | *(103)* |
|  | 2 | 211 | 391 | 63 | 216 |
|  |  | *(150)* | *(297)* | *(21)* | *(108)* |
|  | 3 | 740 | 208 | 43 | 88 |
|  |  | *(436)* | *(159)* | *(39)* | *(77)* |
|  | 4 | 70 | 310 | 72 | 94 |
|  |  | *(37)* | *(223)* | *(33)* | *(48)* |
|  | 5 | 229 | 1498 | 99 | 336 |
|  |  | *(114)* | *(669)* | *(34)* | *(148)* |
|  | 6 | 77 | 380 | 65 | 188 |
|  |  | *(40)* | *(385)* | *(34)* | *(73)* |
|  | 7 | 560 | 233 | 90 | 353 |
|  |  | *(338)* | *(102)* | *(28)* | *(97)* |
|  | 8 | 349 | 663 | 144 | 259 |
|  |  | *(288)* | *(468)* | *(77)* | *(137)* |
|  | 9 | 764 | 775 | 145 | 145 |
|  |  | *(318)* | *(336)* | *(74)* | *(60)* |
|  | 10 | 198 | 37 | 19 | 42 |
|  |  | *(140)* | *(12)* | *(6)* | *(17)* |
|  | 11 | 385 | 1056 | 137 | 137 |
|  |  | *(161)* | *(358)* | *(47)* | *(47)* |
|  | 12 | 850 | 2461 | 279 | 434 |
|  |  | *(319)* | *(846)* | *(103)* | *(133)* |
|  | 13 | 1647 | 672 | 189 | 463 |
|  |  | *(780)* | *(332)* | *(89)* | *(97)* |
|  | 14 | 813 | 1587 | 168 | 907 |
|  |  | *(670)* | *(908)* | *(93)* | *(108)* |
|  | 15 | 1867 | 550 | 187 | 408 |
|  |  | *(1118)* | *(486)* | *(90)* | *(1118)* |
|  | 16 | 2595 | 962 | 95 | 452 |
|  |  | *(1988)* | *(1751)* | *(32)* | *(255)* |
|  | 17 | 3125 | 1509 | 161 | 656 |
|  |  | *(1519)* | *(598)* | *(37)* | *(287)* |
|  | 18 | 616 | 1579 | 334 | 1238 |
|  |  | *(286)* | *(1119)* | *(156)* | *(390)* |
|  | 19 | 417 | 818 | 186 | 536 |
|  |  | *(197)* | *(366)* | *(41)* | *(151)* |
|  | 20 | 1684 | 818 | 374 | 816 |
|  |  | *(842)* | *(493)* | *(168)* | *(368)* |
|  | 21 | 688 | 1268 | 171 | 244 |
|  |  | *(421)* | *(451)* | *(119)* | *(64)* |
|  | 22 | 145 | 497 | 337 | 628 |
|  |  | *(76)* | *(255)* | *(115)* | *(267)* |
|  | 23 | 1011 | 1639 | 167 | 484 |
|  |  | *(729)* | *(474)* | *(49)* | *(151)* |
|  | 24 | 2402 | 3172 | 201 | 815 |
|  |  | *(1219)* | *(1002)* | *(447)* | *(346)* |
|  | 25 | 559 | 634 | 199 | 133 |
|  |  | *(435)* | *(291)* | *(102)* | *(60)* |
|  | 26 | 1137 | 620 | 364 | 340 |
|  |  | *(585)* | *(460)* | *(259)* | *(135)* |
|  | 27 | 833 | 962 | 183 | 270 |
|  |  | *(270)* | *(1059)* | *(24)* | *(56)* |
|  | 28 | 684 | 273 | 605 | 287 |
|  |  | *(765)* | *(293)* | *(106)* | *(139)* |
|  | 29 | 2484 | 1727 | 594 | 503 |
|  |  | *(1274)* | *(1249)* | *(71)* | *(134)* |
|  | 30 | 951 | 962 | 273 | 538 |
|  |  | *(1739)* | *(1781)* | *(102)* | *(63)* |
| **Mean** |  | **951** | **962** | **201** | **408** |
| **SD** |  | ***(820)*** | ***(698)*** | ***(143)*** | ***(275)*** |

**Supplementary Table S2.** Overview of statistical tests in all experimental conditions. Adjusted significance values are reported in bold.

| **APB** | | | | | |  | **FDI** | | | | | |
| --- | --- | --- | --- | --- | --- | --- | --- | --- | --- | --- | --- | --- |
|  | Test statistic | Std. Error | Std. Test Statistic | Sig. | Adj. Sig. |  |  | Test statistic | Std. Error | Std. Test Statistic | Sig. | Adj. Sig. |
| BASELINE VS POST | -.300 | .408 | -.735 | .462 | **1.000** |  | BASELINE VS POST | .167 | .408 | .408 | .683 | **1.000** |
| BASELINE VS AUG-GRASP | -.650 | .408 | -1.592 | .111 | **1.000** |  | BASELINE VS AUG-GRASP | -.800 | .408 | -1.960 | .050 | **.500** |
| BASELINE VS NAT-GRASP | -1.233 | .408 | -3.021 | .003 | **.025** |  | BASELINE VS NAT-GRASP | -.483 | .408 | -1.184 | .236 | **1.000** |
| BASELINE VS PINCH | -1.650 | .408 | -4.042 | <.001 | **.001** |  | BASELINE VS PINCH | -1.967 | .408 | -4.817 | <.001 | **<.001** |
| POST VS AUG-GRASP | .350 | .408 | .857 | .391 | **1.000** |  | POST VS AUG-GRASP | .967 | .408 | 2.368 | .018 | **.179** |
| POST VS NAT-GRASP | .933 | .408 | 2.286 | .022 | **.222** |  | POST VS NAT-GRASP | .650 | .408 | 1.592 | .111 | **1.000** |
| POST VS PINCH | 1.350 | .408 | 3.307 | .001 | **.009** |  | POST VS PINCH | 2.133 | .408 | 5.226 | <.001 | **<.001** |
| AUG-GRASP VS NAT-GRASP | .583 | .408 | 1.429 | .153 | **1.000** |  | AUG-GRASP VS NAT-GRASP | -.317 | .408 | -.776 | .438 | **1.000** |
| AUG-GRASP VS PINCH | 1.000 | .408 | 2.449 | .014 | **.143** |  | AUG-GRASP VS PINCH | 1.167 | .408 | 2.858 | .004 | **.043** |
| NAT-GRASP VS PINCH | .47 | .408 | 1.021 | -307 | **1.000** |  | NAT-GRASP VS PINCH | 1.483 | .408 | 3.633 | <.001 | **.003** |
|  |  |  |  |  |  |  |  |  |  |  |  |  |
| **FDS** | | | | | |  | **EDC** | | | | | |
|  | Test statistic | Std. Error | Std. Test Statistic | Sig. | Adj. Sig. |  |  | Test statistic | Std. Error | Std. Test Statistic | Sig. | Adj. Sig. |
| BASELINE VS POST | .600 | .408 | 1.470 | .142 | **1.000** |  | BASELINE VS POST | .000 | .408 | .000 | 1.000 | **1.000** |
| BASELINE VS AUG-GRASP | -1.717 | .408 | -4.205 | <.001 | **<.001** |  | BASELINE VS AUG-GRASP | -1.583 | .408 | -3.878 | <.001 | **<.001** |
| BASELINE VS NAT-GRASP | -1.400 | .408 | -3.429 | .001 | **.006** |  | BASELINE VS NAT-GRASP | -1.700 | .408 | -4.164 | <.001 | **<.001** |
| BASELINE VS PINCH | -.817 | .408 | -2.000 | .045 | **.455** |  | BASELINE VS PINCH | -.883 | .408 | -2.164 | .030 | **.305** |
| POST VS AUG-GRASP | 2.317 | .408 | 5.675 | <.001 | **<.001** |  | POST VS AUG-GRASP | 1.583 | .408 | 3.878 | <.001 | **.001** |
| POST VS NAT-GRASP | 2.000 | .408 | 4.899 | <.001 | **<.001** |  | POST VS NAT-GRASP | 1.700 | .408 | 4.164 | <.001 | **<.001** |
| POST VS PINCH | 1.417 | .408 | 3.470 | .001 | **.005** |  | POST VS PINCH | .883 | .408 | 2.164 | .030 | **.305** |
| AUG-GRASP VS NAT-GRASP | -.317 | .408 | -.776 | .438 | **1.000** |  | AUG-GRASP VS NAT-GRASP | .117 | .408 | .286 | .775 | **1.000** |
| AUG-GRASP VS PINCH | -.900 | .408 | -2.205 | .027 | **.275** |  | AUG-GRASP VS PINCH | -.700 | .408 | -1.715 | .086 | **.864** |
| NAT-GRASP VS PINCH | -.583 | .408 | -1.429 | .153 | **1.000** |  | NAT-GRASP VS PINCH | -817 | .408 | -2.000 | .045 | **.455** |
|  |  |  |  |  |  |  |  |  |  |  |  |  |
| **HAND** | | | | | |  | **FOREARM** | | | | | |
|  | Test statistic | Std. Error | Std. Test Statistic | Sig. | Adj. Sig. |  |  | Test statistic | Std. Error | Std. Test Statistic | Sig. | Adj. Sig. |
| BASELINE VS POST | .138 | .415 | .332 | .740 | **1.000** |  | BASELINE VS POST | .207 | .415 | .498 | .618 | **1.000** |
| BASELINE VS AUG-GRASP | -.397 | .415 | .-995 | .340 | **1.000** |  | BASELINE VS AUG-GRASP | -1.707 | .415 | -4.111 | <.001 | **<.001** |
| BASELINE VS NAT-GRASP | -.897 | .415 | -2.159 | .031 | **.308** |  | BASELINE VS NAT-GRASP | -1.759 | .415 | -4.235 | <.001 | **<.001** |
| BASELINE VS PINCH | -1.948 | .415 | -4.692 | <.001 | **<.001** |  | BASELINE VS PINCH | -1.052 | .415 | -2.533 | .011 | **.113** |
| POST VS AUG-GRASP | .534 | .415 | 1.287 | .198 | **1.000** |  | POST VS AUG-GRASP | 1.914 | .415 | 4.609 | <.001 | **<.001** |
| POST VS NAT-GRASP | 1.034 | .415 | 2.491 | .013 | **.127** |  | POST VS NAT-GRASP | 1.966 | .415 | 4.734 | <.001 | **<.001** |
| POST VS PINCH | 2.086 | .415 | 5.024 | <.001 | **<.001** |  | POST VS PINCH | 1.259 | .415 | 3.031 | .002 | **.024** |
| AUG-GRASP VS NAT-GRASP | .500 | .415 | 1.204 | .229 | **1.000** |  | AUG-GRASP VS NAT-GRASP | .052 | .415 | .125 | .901 | **1.000** |
| AUG-GRASP VS PINCH | 1.552 | .415 | 3.737 | <.001 | **.002** |  | AUG-GRASP VS PINCH | -.655 | .415 | -1.578 | .115 | **1.000** |
| NAT-GRASP VS PINCH | 1.052 | .415 | 2.533 | .011 | **.113** |  | NAT-GRASP VS PINCH | -.707 | .415 | -1.702 | .089 | **.887** |
|  |  |  |  |  |  |  |  |  |  |  |  |  |
| **AGONIST** | | | | | |  | **NO-AGONIST** | | | | | |
|  | Test statistic | Std. Error | Std. Test Statistic | Sig. | Adj. Sig. |  |  | Test statistic | Std. Error | Std. Test Statistic | Sig. | Adj. Sig. |
| BASELINE VS POST | .103 | .415 | .249 | .803 | **1.000** |  | BASELINE VS POST | .000 | .415 | .000 | 1.000 | **1.000** |
| BASELINE VS AUG-GRASP | -1.224 | .415 | -2.948 | .003 | **.032** |  | BASELINE VS AUG-GRASP | -.776 | .415 | -1.869 | .062 | **.617** |
| BASELINE VS NAT-GRASP | -.966 | .415 | -2.325 | .020 | **.201** |  | BASELINE VS NAT-GRASP | -1.448 | .415 | -3.488 | <.001 | **.005** |
| BASELINE VS PINCH | -2.052 | .415 | -4.941 | <.001 | **<.001** |  | BASELINE VS PINCH | -1.741 | .415 | -4.194 | <.001 | **.001** |
| POST VS AUG-GRASP | 1.328 | .415 | 3.197 | .001 | **.014** |  | POST VS AUG-GRASP | .776 | .415 | 1.869 | .062 | **.617** |
| POST VS NAT-GRASP | 1.069 | .415 | 2.574 | .010 | **.100** |  | POST VS NAT-GRASP | 1.448 | .415 | 3.488 | <.001 | **.005** |
| POST VS PINCH | 2.155 | .415 | 5.190 | <.001 | **<.001** |  | POST VS PINCH | 1.741 | .415 | 4.194 | <.001 | **<.001** |
| AUG-GRASP VS NAT-GRASP | -.259 | .415 | -.623 | .533 | **1.000** |  | AUG-GRASP VS NAT-GRASP | .672 | .415 | 1.619 | .105 | **1.000** |
| AUG-GRASP VS PINCH | .828 | .415 | 1.993 | .046 | **.463** |  | AUG-GRASP VS PINCH | .966 | .415 | 2.325 | .020 | **.201** |
| NAT-GRASP VS PINCH | 1.086 | .415 | 2.616 | .009 | **.089** |  | NAT-GRASP VS PINCH | .293 | .415 | .706 | .480 | **1.000** |

Figure S1. **MEP amplitude changes (percentage versus baseline, expressed as 100%) at single-muscle level**. (a.) APB variation versus resting condition (BASAL-PRE); (b.) FDI variation versus resting condition; (c.) FDS variation versus resting condition; (d.) EDC percentage variation versus resting condition. Bars are standard errors. Note: Percentage variations are used for graphical representations. Significant changes based on raw data statistics between conditions are reported.

Here and in the other two figures, the following parameters are indicated: mean and median values (the cross and the line within the box), the 25^th^ and 75^th^ quartiles (the borders of the box), the 5^th^ and 95^th^ percentiles (the extremes of the whiskers).


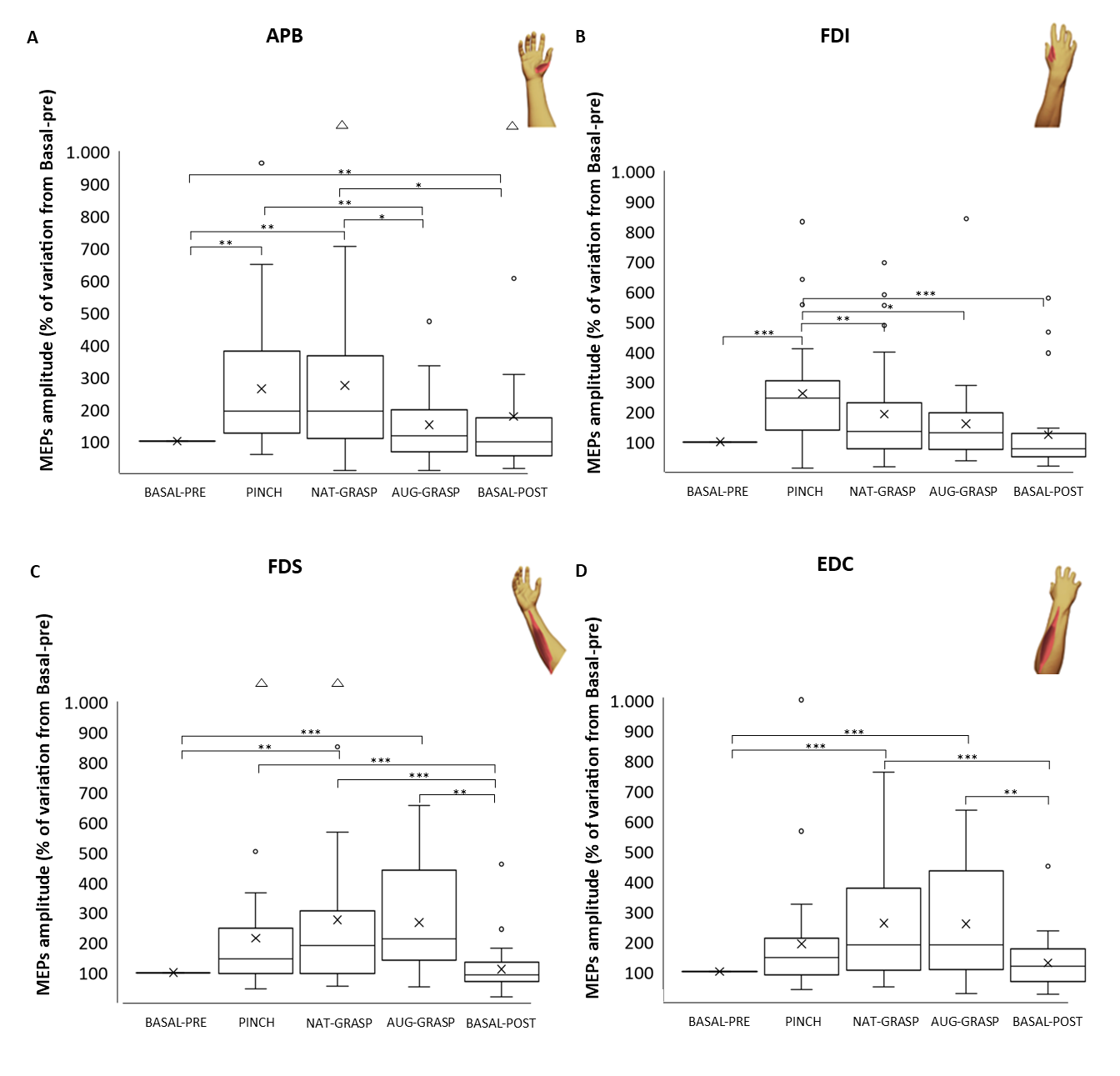


**Figure S2.** **Intrinsic (“Hand”) versus extrinsic (“Forearm”) muscles effects.** MEPs amplitude modulation (percentage variation versus Baseline, expressed as 100%) versus Basal-Pre condition of distal HAND (FDI+APB) versus proximal FOREARM (EDC+FDS) muscle. Bars are standard errors. Note: Percentage variations are used for graphical representations. All significant changes are reported.


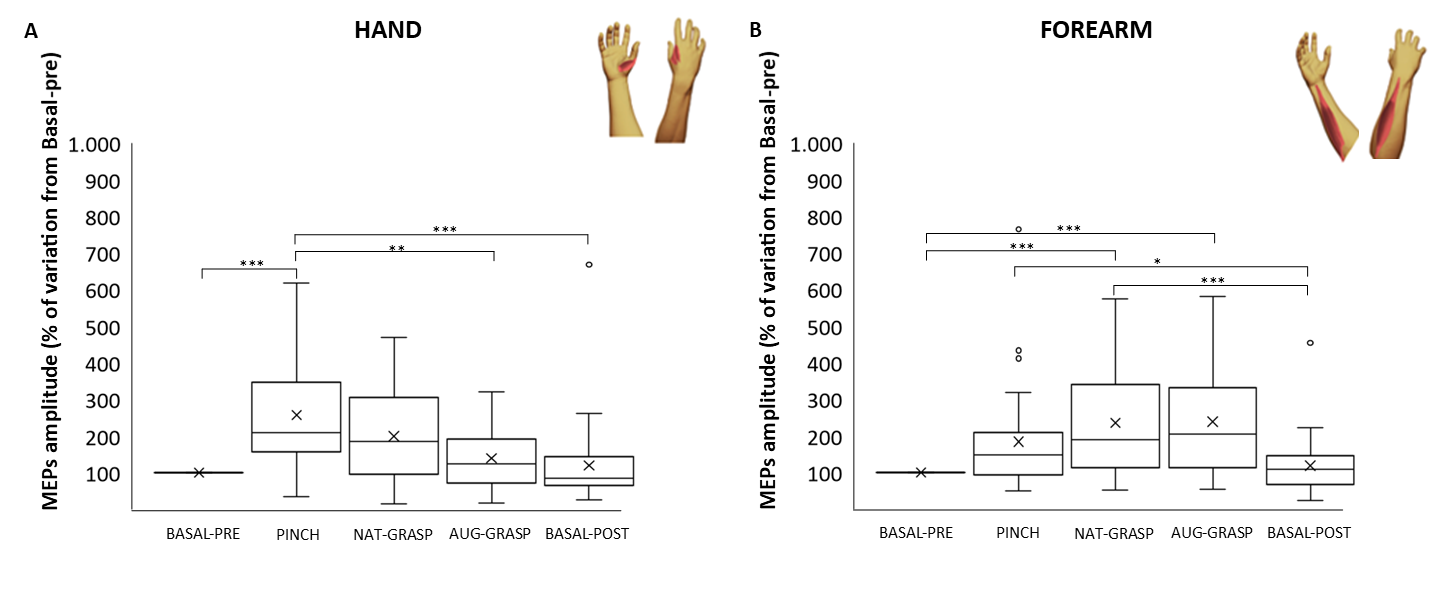


**Figure S3.** **Agonist (FDS+FDI) versus non-agonist (EDC+APB) muscles MEPs amplitude (Baseline is 100%)**. (a) agonist muscles percentage variation versus resting condition (BASAL-PRE); (b.) Non-agonist muscles percentage variation versus resting condition (BASAL-PRE). Bars are standard errors. Note: Percentage variations are used for graphical representations. All significant changes are reported.


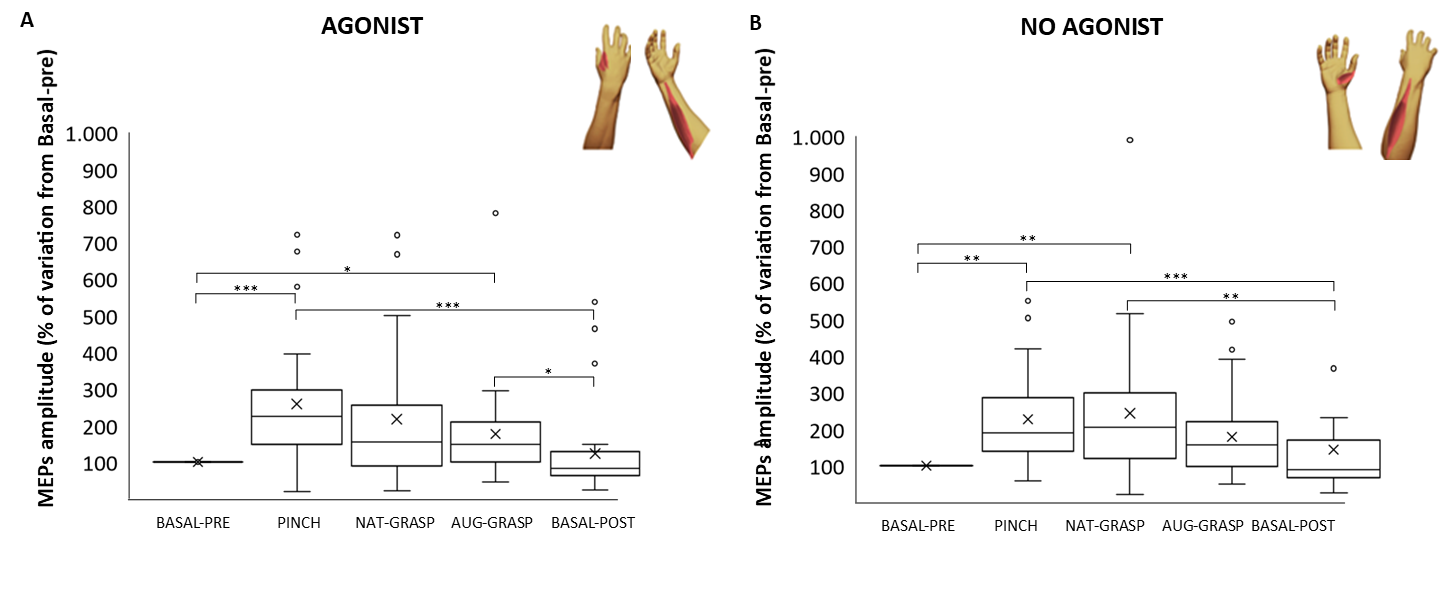

Supplement: Supplementary file 1 — Supplementary Information. [file 41598_2021_97876_MOESM1_ESM.docx]
